# Development of the arterial roots and ventricular outflow tracts

**DOI:** 10.1111/joa.13973

**Published:** 2023-11-13

**Authors:** Robert H. Anderson, Wouter H. Lamers, Jill P. J. M. Hikspoors, Timothy J. Mohun, Simon D. Bamforth, Bill Chaudhry, Lorraine Eley, Janet Kerwin, Moira Crosier, Deborah J. Henderson

**Affiliations:** ^1^ Biosciences Institute Newcastle University Newcastle upon Tyne UK; ^2^ Department of Anatomy & Embryology Maastricht University Maastricht The Netherlands; ^3^ Crick Institute London UK

**Keywords:** anatomy, arterial valves, intrapericardial arterial trunks, outflow myocardium

## Abstract

The separation of the outflow tract of the developing heart into the systemic and pulmonary arterial channels remains controversial and poorly understood. The definitive outflow tracts have three components. The developing outflow tract, in contrast, has usually been described in two parts. When the tract has exclusively myocardial walls, such bipartite description is justified, with an obvious dogleg bend separating proximal and distal components. With the addition of non‐myocardial walls distally, it becomes possible to recognise three parts. The middle part, which initially still has myocardial walls, contains within its lumen a pair of intercalated valvar swellings. The swellings interdigitate with the distal ends of major outflow cushions, formed by the remodelling of cardiac jelly, to form the primordiums of the arterial roots. The proximal parts of the major cushions, occupying the proximal part of the outflow tract, which also has myocardial walls, themselves fuse and muscularise. The myocardial shelf thus formed remodels to become the free‐standing subpulmonary infundibulum. Details of all these processes are currently lacking. In this account, we describe the anatomical changes seen during the overall remodelling. Our interpretations are based on the interrogation of serially sectioned histological and high‐resolution episcopic microscopy datasets prepared from developing human and mouse embryos, with some of the datasets processed and reconstructed to reveal the specific nature of the tissues contributing to the separation of the outflow channels. Our findings confirm that the tripartite postnatal arrangement can be correlated with the changes occurring during development.

## Graphical abstract

Labelling of cardiac muscle and cushion cells in the pulmonary root in the human and mouse heart at comparable stages of development.

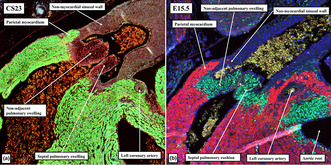

## 1 INTRODUCTION

In one of the seminal accounts of cardiac development, Kramer suggested that the outflow tract could best be assessed in terms of two parts, namely the conus and the truncus (Kramer, 1942). This concept, although well accepted, suffers a significant flaw. The bipartite approach fails to provide the details of the formation of the arterial roots. These entities, key components of the outflow tracts, are located between the intrapericardial arterial trunks, believed to develop from the “truncus” of Kramer, and the ventricular outflow tracts, usually interpreted as representing the “conus.” The commonest congenital cardiac malformation, however, namely the bicuspid aortic valve (Roberts, 1984), is not currently recognised as a “conotruncal” malformation (Van Praagh, 2016). In his account, Kramer suggested that “a redefinition of the terms used in describing the structures involved has proved urgently necessary, both because of the discrepant terms employed by previous workers, and also because of the rapidly changing shapes and locations in which the structures themselves are found at different stages of development” (Kramer, 1942). The latter part of his statement retains its validity. It explains why we still remain uncertain as to how, precisely, the outflow tract changes from its initial form, as seen subsequent to formation of the ventricular loop, to the definitive postnatal arrangement.

If the outflow tract is defined as the part of the heart tube extending from the developing right ventricle to the margins of the pericardial cavity, when first formed it has exclusively myocardial walls. This is the situation in both the mouse and human embryo (Anderson et al., 2012; Sizarov et al., 2012). In the postnatal situation, in contrast, the greater parts of the walls of the outflow channels are arterial. The myocardial components are restricted to the free‐standing myocardial infundibulum of the right ventricle, which includes crescentic areas incorporated at the bases of all three sinuses of the pulmonary root (Mori et al., 2019), and comparable myocardial crescents found at the bases of the sinuses of the aortic root which give rise to the coronary arteries (Toh et al., 2020). This means that continued development must involve a change in the relationship between the distal myocardial border and the margins of the pericardial cavity. Some of this change may involve differential growth of the original tissues. The tissues separating the developing arterial roots, however, are initially mesenchymal, being formed from the fused major outflow cushions. With ongoing development, parts of these cushions undergo a process of myocardialisation (van den Hoff et al., 1999; van den Hoff & Wessels, 2020). As had been predicted by Kramer (1942), it is indeed the changing shapes and locations of the myocardial, as opposed to the non‐myocardial, components of the outflow tracts that provide the basis for understanding their development. We provide here an account of these processes.

## 2 NOTES ON THE MATERIALS EXAMINED FOR THIS ANATOMICAL INVESTIGATION

For the purposes of our investigation of the changes taking place in the developing human heart during the embryonic period, we utilised a range of existing material, including inferences drawn from analysis of the interactive pdf files of 3D reconstructions of human developing hearts described in Hikspoors et al. (2022). For the specific purposes of this investigation, and to supplement these reconstructions, we analysed serially sectioned datasets mostly stained with haematoxylin and eosin, but with some processed to show the extent of the myocardium. The datasets were prepared from human embryos from the Human Developmental Biology Resource (HDBR; https://hdbratlas.org; Gerelli et al., 2015). They included specimens obtained during late embryonic and the early fetal period of development. We also assessed additional existing datasets from human embryos prepared using episcopic microscopy (Mohun & Weninger, 2012), with the specimens having been obtained originally from HDBR (Table 1). We then compared the findings from the human embryos with multiple histological and episcopic datasets from developing mouse embryos and fetuses (Table 1). Many of these have been described previously (Anderson et al., 2015; Mohun & Anderson, 2020). Large numbers of embryos and fetuses were prepared using episcopic microscopy, with some obtained from XMlc2‐Cre (Breckenridge et al., 2007) and Isl1‐Cre (Cai et al., 2003) mice. Other datasets had been prepared by serial histological sectioning (Richardson et al., 2018). Some episcopic datasets were analysed and reconstructed specifically for this investigation using Amira software (Anderson et al., 2015; Mohun & Anderson, 2020).

**TABLE 1:** Specimens examined.

Human hearts	HREM	Histologic	Mouse hearts	HREM	XLMC2Cre	Islet1Cre
Carnegie stage 11	0	3	Embryonic day 10.5	17	0	0
Carnegie stage 12	1	2	Embryonic day 11.5	31	2	0
Carnegie stage 13	3	4	Embryonic day 12.5	12	6	0
Carnegie stage 14	5	8	Embryonic day 13.5	11	2	0
Carnegie stage 15	4	3	Embryonic day 14.5	8	3	4
Carnegie stage 16	3	10	Embryonic day 15.5	12	1	2
Carnegie stage 17	1	6	Embryonic day 16.5	2	0	0
Carnegie stage 18	0	4	Embryonic day 17.5	3	0	0
Carnegie stage 19	0	3	Embryonic day 18.5	9	0	0
Carnegie stage 20	1	1				
Carnegie stage 21	0	4				
Carnegie stage 22	0	1				
Carnegie stage 23	0	2				

*Note*: The numbers show the specimens examined for each of the stages. All the episcopic (HREM) datasets, including the Cre preparations, were prepared by Dr Mohun, initially at the National Institute of Medical Research, and subsequently at the Crick Institute. The histological datasets were provided by the Human Developmental Biology Resource.

For new datasets prepared specifically for this study, C57Bl/6 mice were used, being maintained according to the Animals (Scientific Procedures) Act 1986, United Kingdom, under project licenses PPL 30/3876 and P9E095FF4. All experiments were approved by the Newcastle University Ethical Review Panel. The methodology has been published previously (Phillips et al., 2013). Antibodies utilised were Sox9 (Abcam; ab185230), cardiac Troponin I (HyTest; 4T21/2) and alpha‐smooth muscle actin (Abcam; ab5228 and ab5694).

## 3 RESULTS

### 3.1 Initial formation of the outflow tract

**FIGURE 1:**
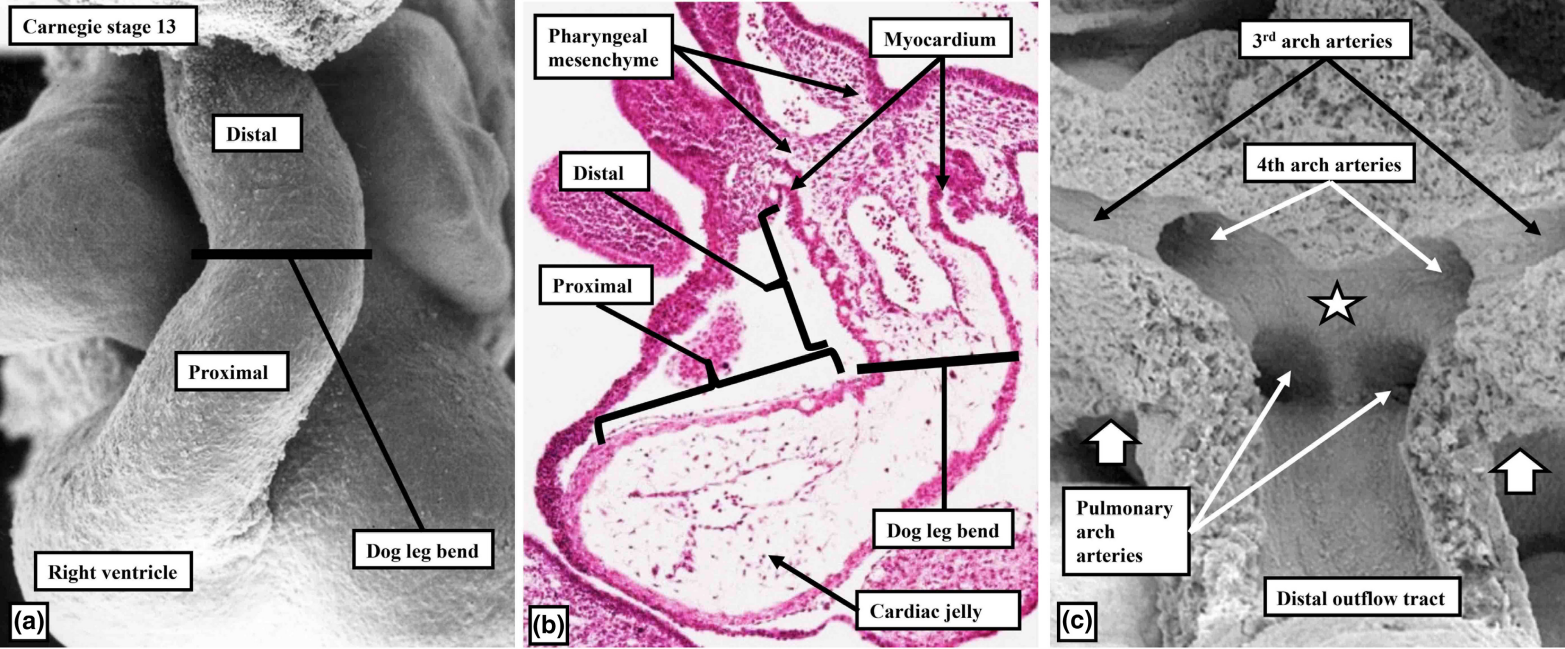
The images show the arrangement of the outflow tract in the human heart at Carnegie stage 13, when around 32 days have elapsed subsequent to fertilisation. Panels (a) and (c) show scanning electron micrographs. The original images were prepared by the late Gerd Steding, working with the team from the Department of Anatomy and Embryology of the University of Amsterdam, and are modified and reproduced with the permission of Joerg Maenner and Mrs Steding. Panel (c) was prepared by removing the anterior wall of the pharyngeal mesenchyme. The star shows the dorsal wall of the aortic sac, which protrudes to become the aortopulmonary septum, while the white stars with black borders show the margins of the pericardial cavity. Panel (b) is an H&E section.

At Carnegie stage (CS) 13 in human development, representing approximately 32 days subsequent to fertilisation, the ventricular component of the developing heart has undergone the process described as looping. By this stage, the apical parts of the ventricles have begun to balloon from the outer curvature of the loop. This makes it possible to recognise the developing primordiums of the right and left ventricles (Hikspoors et al., 2022). The comparable arrangement is seen at embryonic day (E) 10.5 in mouse embryos. In both species at these stages, the outflow tract is a tube with a solitary lumen. It extends from the developing right ventricle to the margins of the pericardial cavity (Figure 1a). Its walls at this stage are exclusively myocardial (Figure 1b; Anderson et al., 2012; Sizarov et al., 2012). At the margins of the pericardial space, the cavity of the outflow tract becomes continuous with that of the manifold found within the pharyngeal mesenchyme known as the aortic sac (Figure 1c). Throughout the stages we have investigated, we have then used the margins of the pericardial cavity to provide the anatomical boundary between the intrapericardial and extrapericardial components of the outflow tract. At the initial stage of development, as shown in Figure 1b, the distal myocardial border is confluent with the margins of the pericardial cavity (Anderson et al., 2012).

### 3.2 The appearance of non‐myocardial tissues within the outflow tract

**FIGURE 2:**
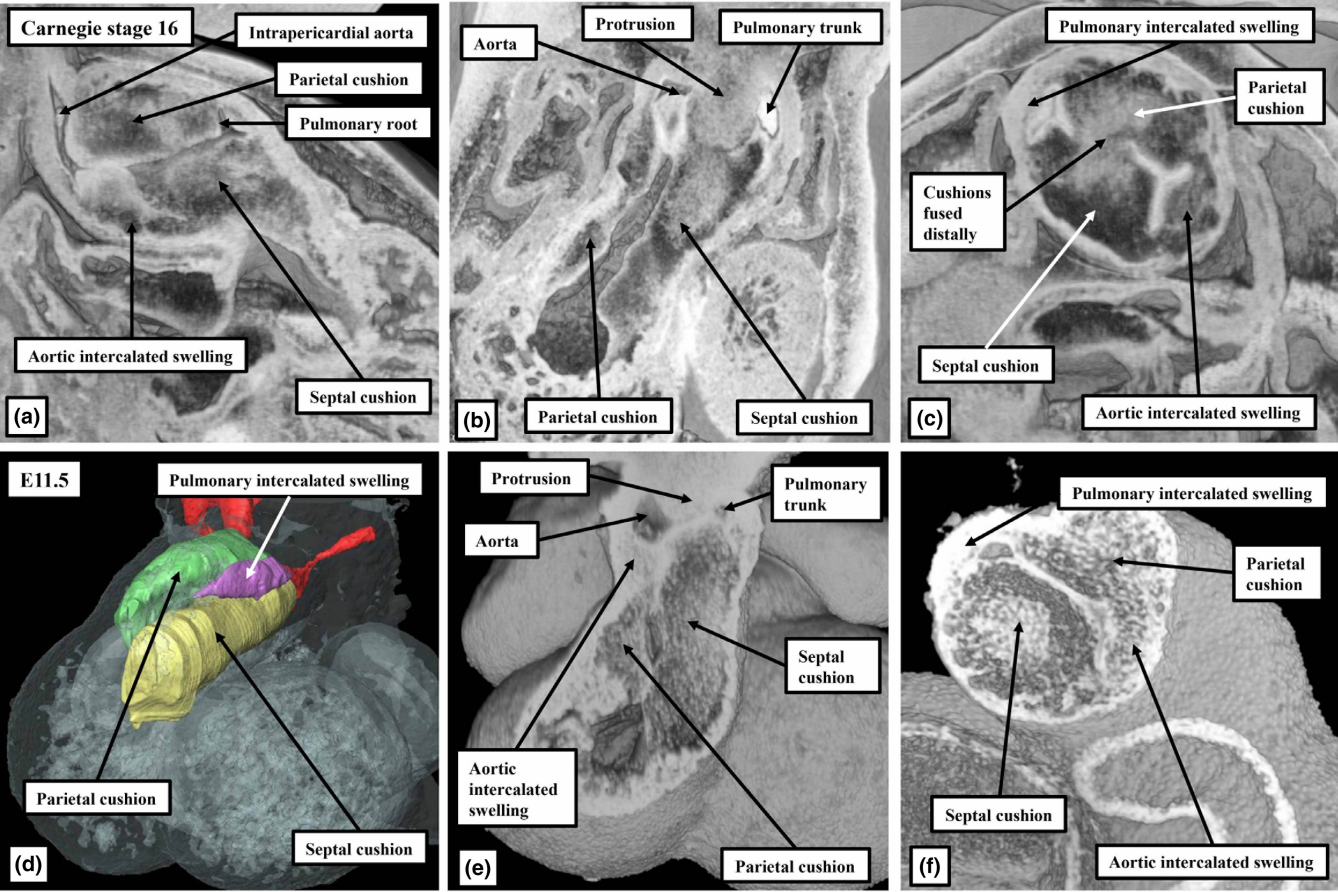
The images are prepared from episcopic datasets made from a CS 16 human embryo (upper panels), and from a E11.5 mouse embryo. Panels (a)–(c) show orthogonal sections through the human dataset, while panel (d) shows the reconstructed cushions and swellings in the mouse dataset, with panels (e) and (f) showing orthogonal sections. The images show the formation of the non‐myocardial distal part of the outflow tract and the appearance of the intercalated valvar swellings.

**FIGURE 3:**
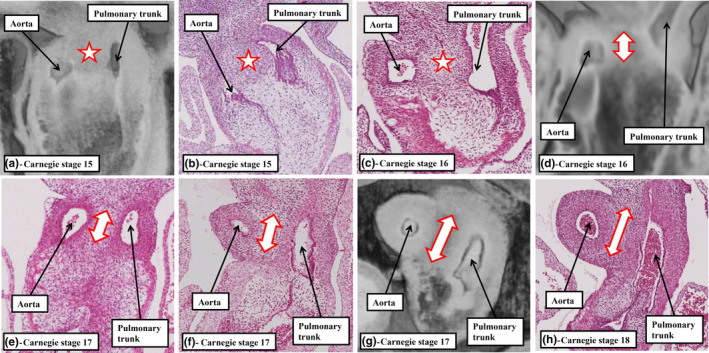
The images are prepared from episcopic datasets (panels a, d, and g) and histological sections (panels b, c, e, f, and g) showing how the core of the protrusion from the dorsal wall of the aortic sac (white star with red borders in panels (a) through (c)) is gradually transformed into an area of tissue that no longer separated the cavities of the developing intrapericardial aorta and pulmonary trunk (double‐headed arrows in panels (d)–(g)). Instead, concomitant with the formation of the adjacent walls of the arterial trunks, the tissue forms an extracavitary area, and no longer fulfils a septal function. All the images are from human embryos during the stages CS15 through CS18.

**FIGURE 4:**
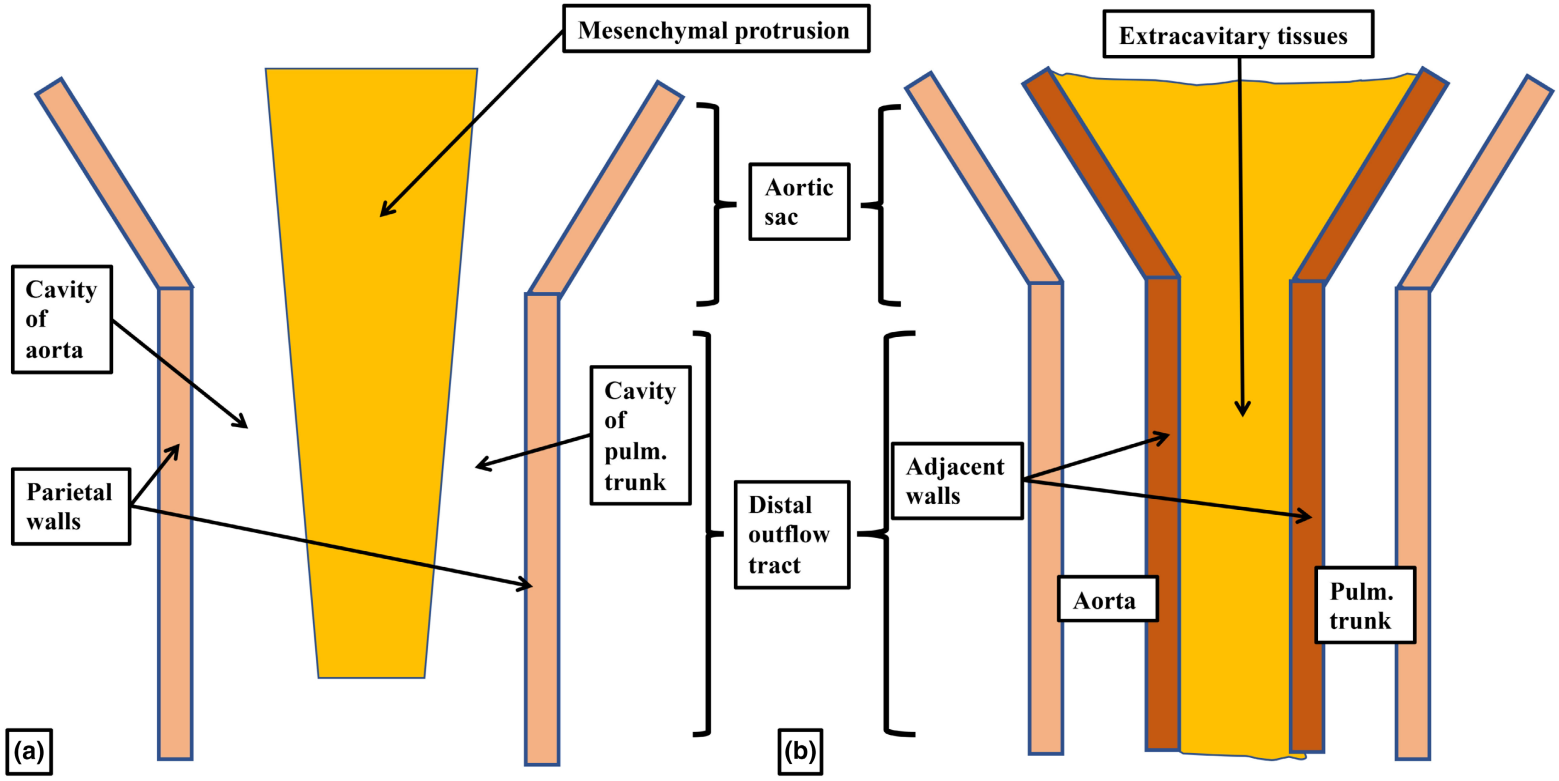
The drawings illustrate the change that takes place as mesenchymal tissue masses lose their septal function. Panel (a) shows the arrangement in which, at the initial stage, the protrusion from the dorsal wall of the aortic sac has entered the cavity of the distal outflow tract. The boundary between the walls of the sac and the distal outflow tract is formed by the pericardial reflections. At this stage, the protrusion is an aortopulmonary septum, since it interposes between the developing aortic and pulmonary components of the cavity. In panel (b), we show the stage at which the protrusion has fused with the distal margins of the outflow cushions. The formation of the adjacent walls of the intrapericardial arterial trunks means that the central part of the mesenchymal mass now interposes between the separate walls. It is now an area of extracavitary tissue, and no longer functions as a septum.

A key feature of ongoing development, in both the human and mouse hearts, is the addition of non‐myocardial tissues from the second heart field to the intrapericardial part of the outflow tract. By virtue of the formation of the new non‐myocardial walls, the distal myocardial border no longer retains its location at the level of the pericardial boundaries. In anatomical terms, therefore, when assessed relative to these boundaries, the myocardial border can be considered to have moved proximally. During the same period of development, the dorsal wall of the aortic sac protrudes into the cavity of the newly formed, non‐myocardial, distal outflow tract. In so doing, it forms an embryonic aortopulmonary septum (Anderson et al., 2012). During the initial period, when the distal walls had been exclusively myocardial, nascent circumferential endocardial cushions had lined its cavity (Figure 1b; Anderson et al., 2012; Phillips et al., 2013). By the time the distal component has developed its non‐myocardial walls (Figure 2a,b,e), the cellularising tissues have remodelled to form two cushions within the part retaining its myocardial walls (Figure 2c). During this period, although the tract itself has straightened (Figure 2), the newly formed cushions spiral relative to one another within its lumen. One of the cushions has a septal location proximally but turns inferiorly beneath the other cushion when traced distally, with the other cushion having a parietal proximal location (Mohun & Anderson, 2020; Figure 2d). By CS16, which is during the sixth week of human gestation, equivalent to E12.5 in the mouse, it has also become possible to recognise the primordiums that will form the arterial roots (Figure 2c,f). This is because, by this time, swellings have formed at the proximal ends of the parietal tongues of non‐myocardial distal outflow tract wall (Figure 2; Anderson et al., 2012). Each of these swellings, named by Kramer as the intercalated valve swellings I (Kramer, 1942), abuts on its luminal surface against the unfused parietal margins of the two major cushions. To complete this stage of development, the distal tips of the main cushions, still within their sleeve of myocardium, fuse not only with each other but also with the protrusion from the dorsal wall of the aortic sac (Figure 2b,e; Anderson et al., 2012). This process results in separation of the distal non‐myocardial component into the intrapericardial arterial trunks. When initially identifiable as a protrusion, the tissue itself is a relatively homogeneous mass of mesenchyme (Figure 3a,b). Between Carnegie stages 15 and 16 in the developing human heart, the margins of the mass begin to become converted to the adjacent walls of intrapericardial arterial trunks (Figure 3c,d). With increasing development of the arterial walls, the central component of the mesenchymal tissue mass no longer interposes between the developing cavities of the arterial trunks. Instead, it becomes an area of tissue interposing between the newly formed arterial walls (Figure 3e–h). At the beginning of these changes, the protrusion had functioned as an aortopulmonary septum, separating the cavity of the distal outflow tract into aortic and pulmonary components (Figure 4a). With the subsequent development of the adjacent walls of the intrapericardial arterial trunks, producing separate vascular channels, the central part of the protrusion is changed into an area of extracavitary space. The protrusion, therefore, has ceased to be a septum (Figure 4b). A comparable process of formation of extracavitary tissues from initial embryonic septal structures is subsequently found throughout the developing middle and proximal parts of the outflow tracts.

### 3.3 Initial changes within the middle part of the outflow tract

**FIGURE 5:**
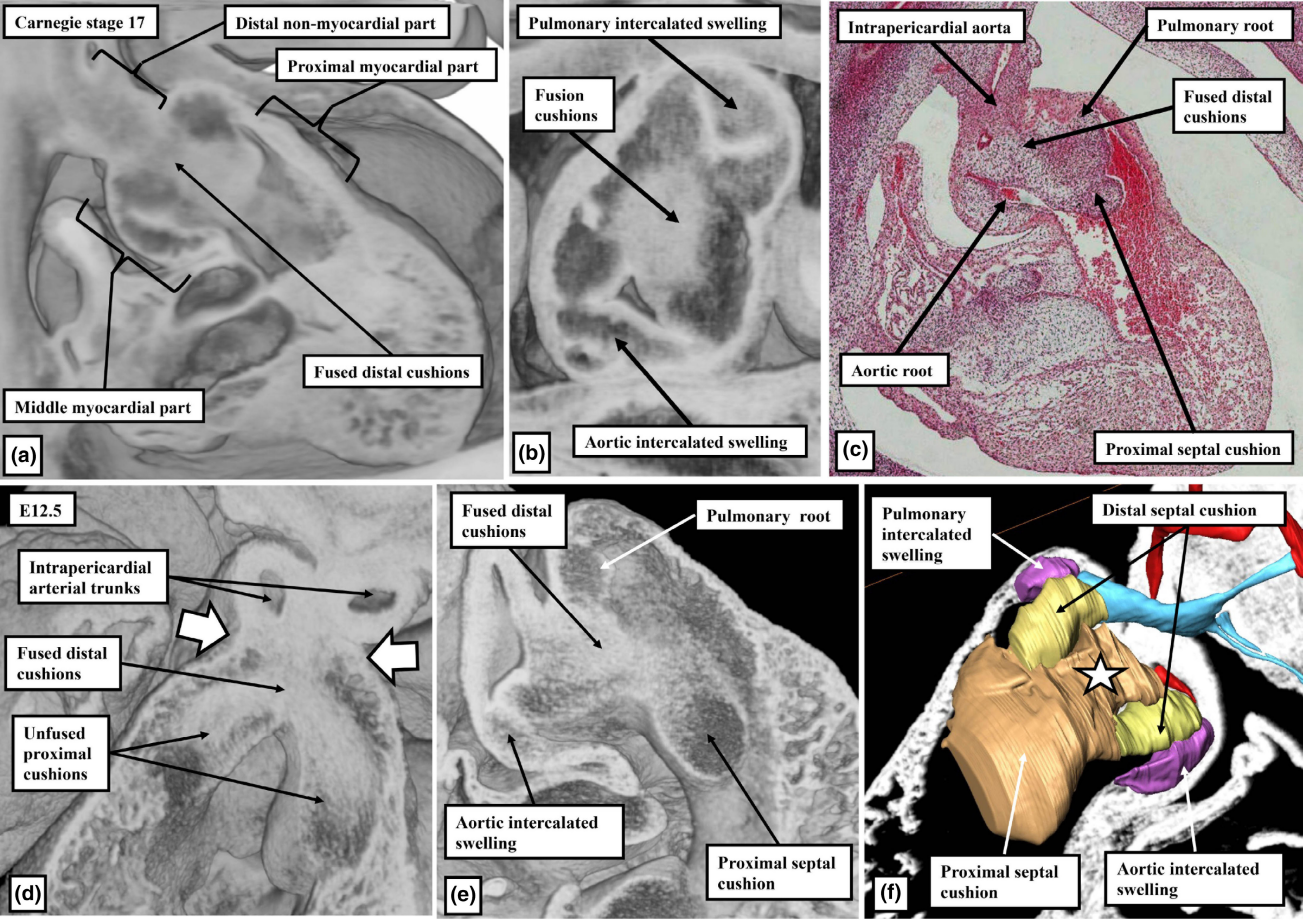
The images are from a CS17 human (a–c) and E12.5 mouse (d–f) datasets. Panels (a) and (b), along with panels (d)–(f), are all from episcopic datasets, with the main outflow cushions and intercalated valve swellings reconstructed in panel (f). The star shows the central core of the fused distal cushions, with the intercalated valve swellings shown in purple, and the unfused parietal ends of the fused septal cushions shown in yellow. The white arrows with black borders in panel (d) show the boundary between the distal and middle parts of the outflow tract. Panel (c) is an H&E section, again from a CS17 human embryo. www.hdbratlas.org. In panel (f), purple shows the intercalated valvar swellings, with yellow showing the distal margins of the major outflow cushions. Brown shows the fused proximal cushion, with the star showing the distal part of the fused distal cushions.

As part of the changes that produced the separate intrapericardial arterial trunks, the boundary between the non‐myocardial walls and the distal myocardial walls of the outflow tract, which initially presented a fishmouth configuration (Anderson et al., 2012; Hikspoors et al., 2022), has become annular. The circular border thus formed is distant when assessed relative to the pericardial boundaries. The extent of the intercalated valve swellings can now be used to identify the distal and proximal boundaries of the middle part of the outflow tract. The tripartite arrangement, with a distal non‐myocardial component, and middle and proximal myocardial parts, can be recognised by CS17 in the developing human heart, approximately 40 days post‐conception (Figure 5a), and at E12.5 in the mouse (Figure 5d). The myocardium surrounding the middle part encases the arterial roots and their forming valves (Figure 5c,f). By this stage, the primordiums of the roots themselves have been separated one from the other by the fused central parts of the distal outflow cushions (Figure 5b,d,e). The more proximal parts of the cushions, which occupy the proximal part of the outflow tract, which also has myocardial walls, have still to fuse. Subsequent to their fusion, these cushions will muscularise to become the free‐standing subpulmonary infundibulum (Figure 5d,e). The arrangements are again comparable in the hearts of humans and mice (Figure 5). The intrapericardial aorta and pulmonary trunk, by this stage, have developed their own discrete walls. As already emphasised, with the formation of the separate walls, the area initially occupied by the core of the embryonic aortopulmonary septum has been converted to an area of extracavitary tissue, occupying the space between the newly formed walls of the intrapericardial arterial trunks, rather than separating their cavities (Figures 3d–g and 4).

### 3.4 Separation of the arterial roots and formation of the infundibulum

**FIGURE 6:**
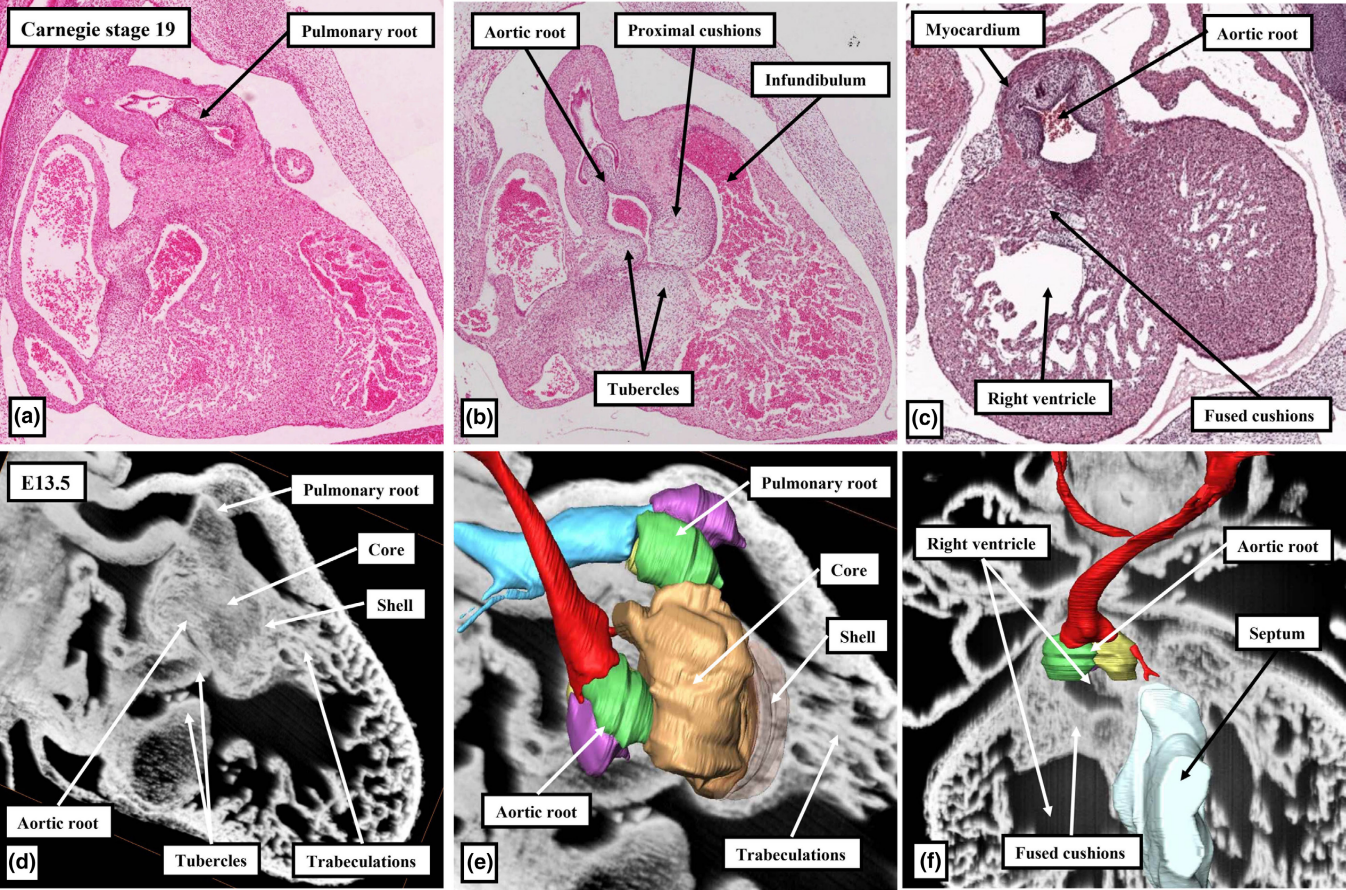
The histological sections, shown in panels (a)–(c), stained with haematoxylin and eosin, are from CS19 human sectioned in the sagittal (a, b) and frontal (c) plane (www.hdbratlas.org). The images in panels (d)–(f) are all from the same episcopic dataset from a E13.5 mouse embryo. The cushions and swellings have been reconstructed in panel (e), with the green colour now showing the distal ends of the parietal cushion. The brown colour shows the core of the fused cushion mass. The purple again shows the intercalated swellings. In panel (f), the aortic root has been reconstructed, along with the muscular ventricular septum. The fused cushions have produced a shelf at the base of the ventricle that incorporates a part of the cavity of the right ventricle within the aortic root.

**FIGURE 7:**
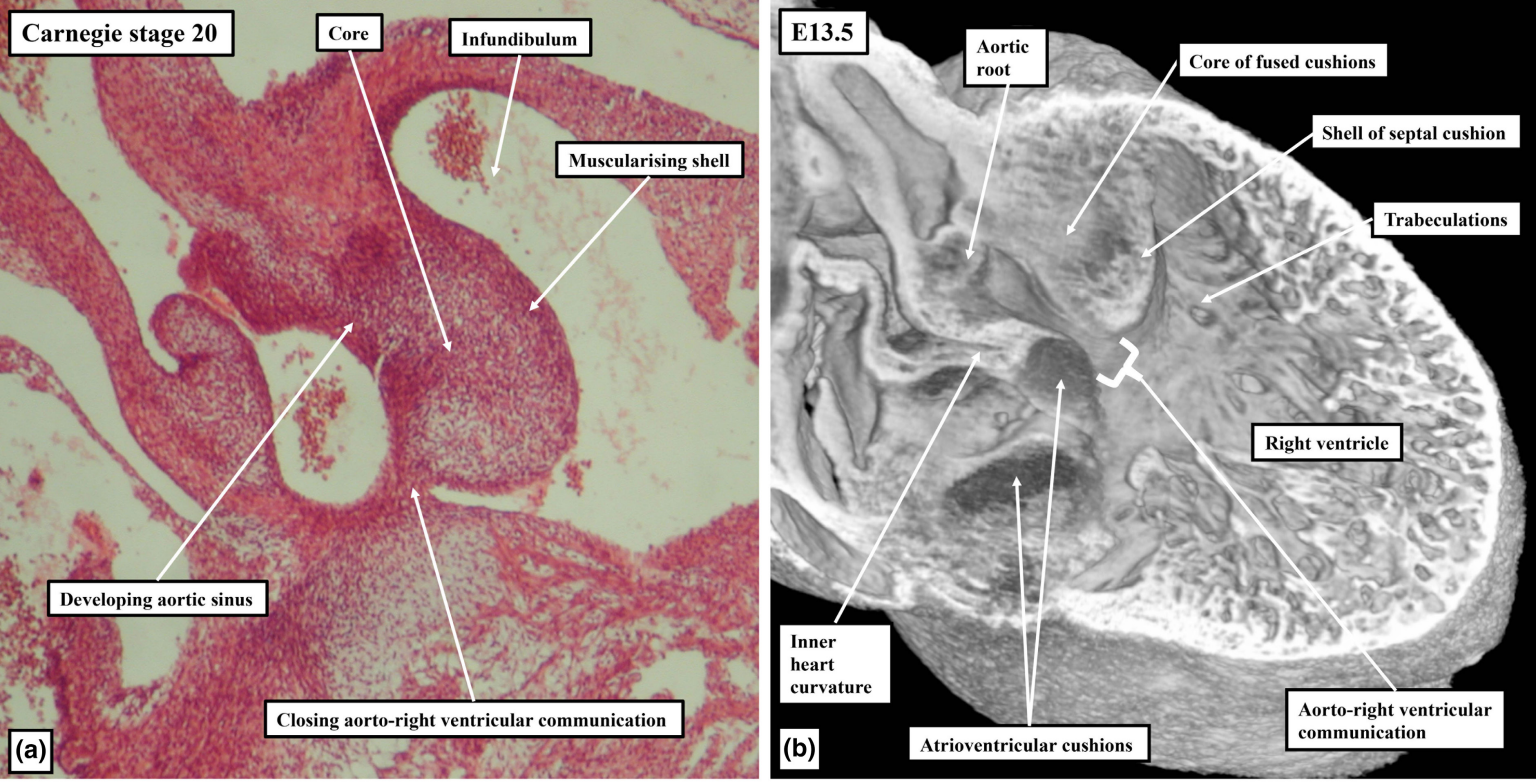
The images, which show closure of the aorto‐right ventricular communication by fusion between the so‐called tubercles of the atrioventricular cushions and the muscularising proximal outflow cushions, compare a sagittal histological section from a human embryo at CS 20 and a sagittal cut through an episcopic dataset prepared from an E13.5 mouse embryo.

**FIGURE 8:**
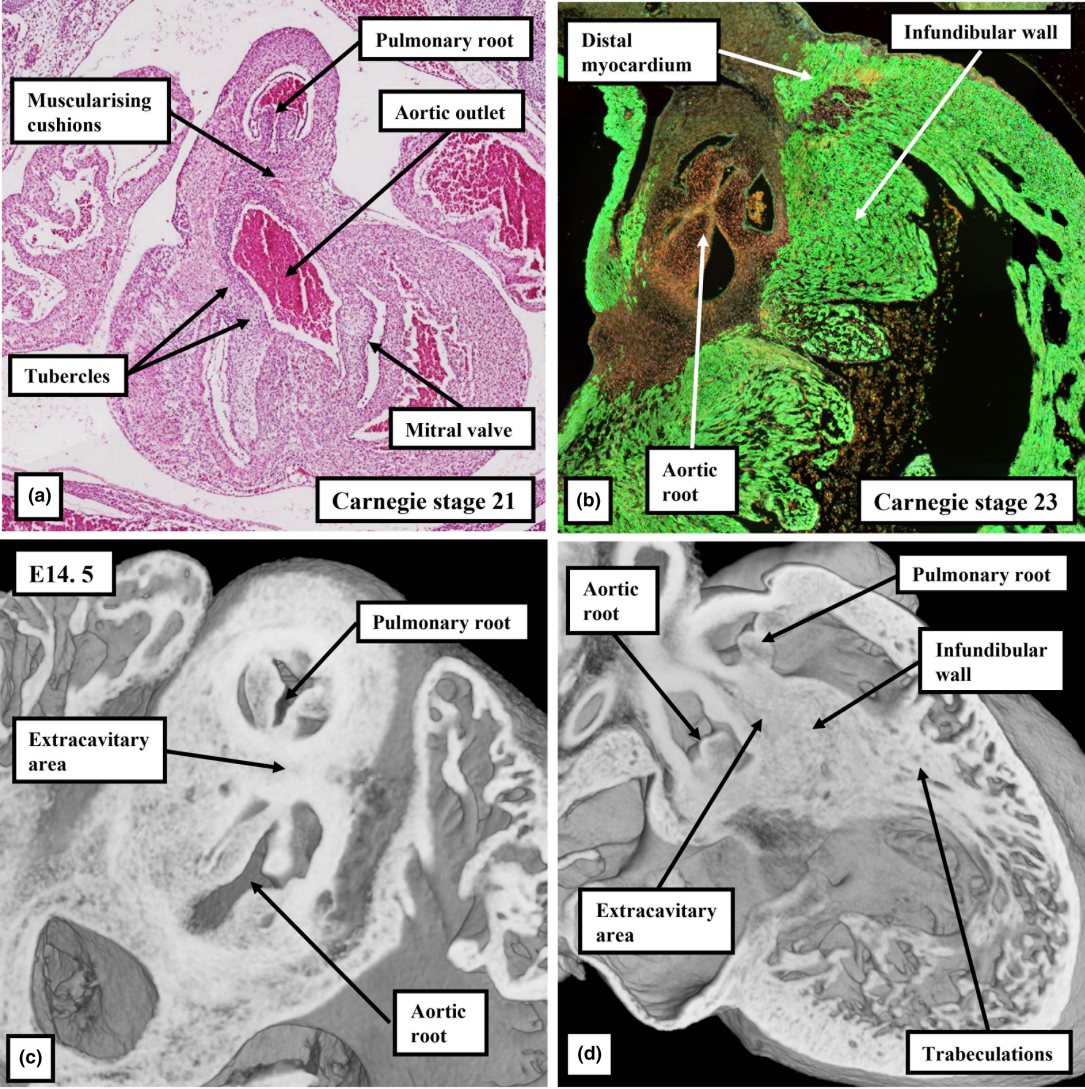
The images show, in panels (a) and (c), the arrangement of the arterial roots at the completion of ventricular septation, and in panels (b) and (d) the muscularisation of the proximal outflow cushions to form the free‐standing subpulmonary infundibulum. The upper panels show sections from CS 21 embryos (in (b), green cTnI, red: Sox9). The lower panels are from an E13.5 episcopic dataset; panel (c) shows a short axis section viewed from above, while panel (d) shows a sagittal section of the right ventricle and aortic root.

**FIGURE 9:**
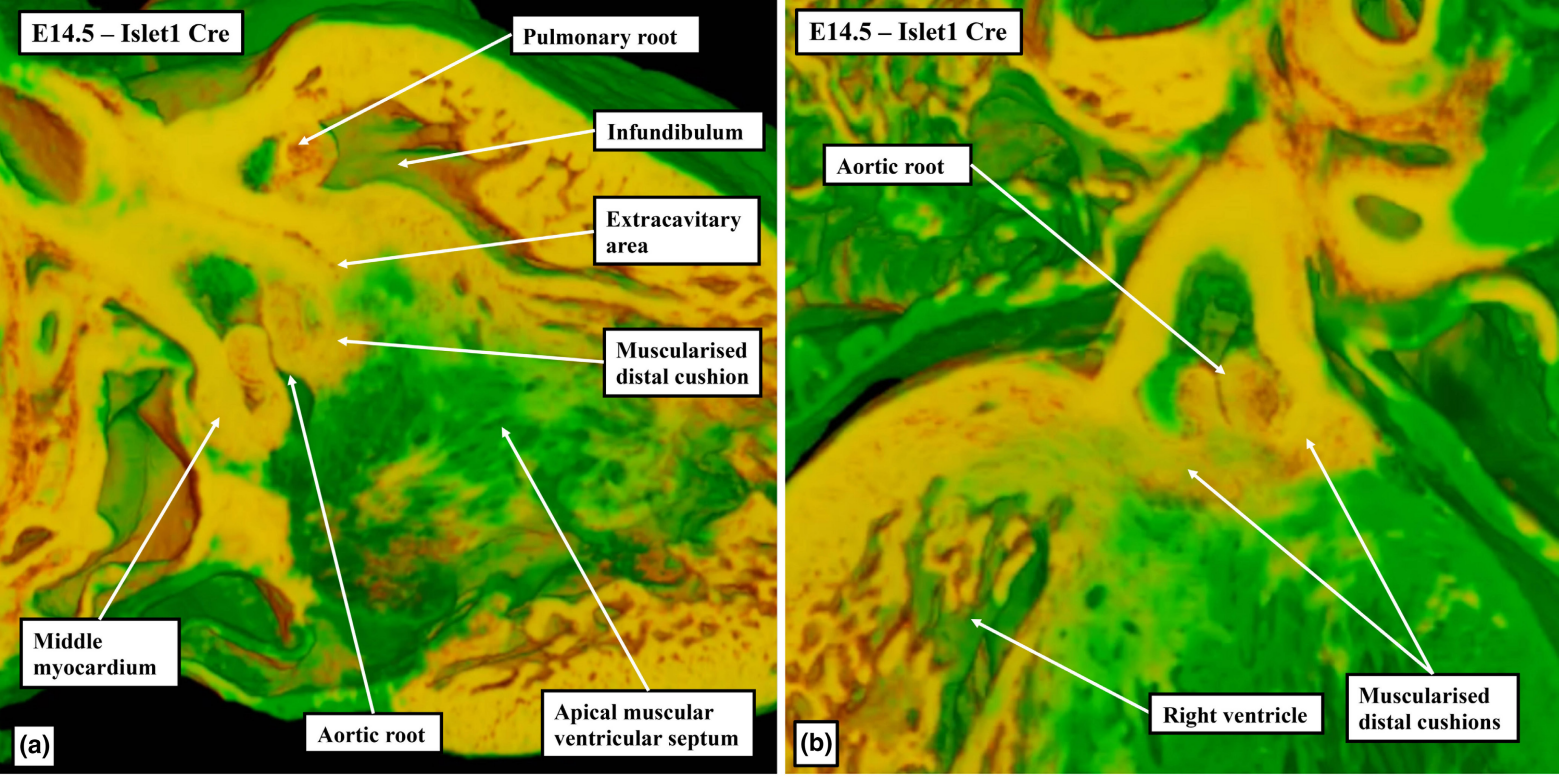
The images are taken from the same episcopic dataset prepared from a E14.5 Isl1Cre+ mouse embryo; the yellow shows cells that are Isl1‐Cre+ and thus are derived from the second heart field (SHF). Panel (a) is a sagittal cut through the ventricular septum, with the larger part of the septum being unstained, showing it is not derived from the SHF. Panel (b) is a frontal cut, showing the Isl1‐Cre+ tissue is confined to the aortic root and is not found in the left ventricle, which is not labelled.

The next stage of development is heralded by the fusion of the proximal parts of the major cushions. This initially creates an arch at the base of the right ventricle (Figure 5e). Fusion is complete by CS19 in the human heart, which is towards the end of the seventh week after conception, and by the end of E13.5 in the developing mouse embryo (Figure 6). With the completion of fusion, the arch initially produced within the right ventricle has been converted into a partition, thus sequestering a small part of the right ventricular cavity beneath the aortic root (Figure 6c,f). The aortic root itself at this stage, however, is still exclusively supported by the walls of the developing right ventricle. It communicates with the cavity of the left ventricle through the channel best described as the secondary interventricular foramen. The initial, or primary, interventricular foramen is seen at the earlier stages where the atrioventricular canal opens exclusively to the developing left ventricle, and the developing right ventricle supports the entirety of the cavity of the outflow tract (Mohun & Anderson, 2020). At the dorsal extent of the newly formed partition, nonetheless, it retains a smaller communication with the larger apical cavity of the right ventricle (Figure 7). This aortic‐right ventricular channel can be considered to represent the tertiary interventricular foramen (Mohun & Anderson, 2020) The inferior surface of the partition formed by fusion of the proximal cushions, which faces the major part of the right ventricular cavity, has by now begun to muscularise (van den Hoff et al., 1999; van den Hoff & Wessels, 2020). The core of the fused cushion mass, including its distal component, in contrast, does not muscularise. This central part contains cells derived from the neural crest. These cells could be recognised in histological sections at earlier stages as the areas of condensed mesenchyme which formed a so‐called whorl at the site of distal fusion with the aorto‐pulmonary septum and continued as prongs extending proximally within the cushions (Figure 3d). The non‐muscularised core of the cushions, initially quite substantial, separates the muscularising shell forming on the rightward wall of the fused cushions from the developing aortic root, which is positioned on the left side of the cushion mass (Figure 6d,e). The shell of the shelf is attached to the crest of the muscular ventricular septum. The attachment is between the trabeculations of the right ventricle, which themselves are coalescing to reinforce the right ventricular surface of the muscular septum as the septomarginal trabeculation, or septal band (Figure 6e). As was explained above, on the basis that the subaortic outflow tract is destined to become part of the cavity of the left ventricle, the communication existing between the aortic root and the apical part of the right ventricle (Figure 7) can also be considered to represent a tertiary interventricular communication (Anderson et al., 2014; Mohun & Anderson, 2020). The opening is bounded cranially by the muscularising attachment of the septal cushion, and caudally by the rightward margins of the fusing atrioventricular cushions (Figure 7b). The tips of these rightward margins, which were described initially as tubercles (Odgers, 1938), become transformed, subsequent to the closure of the tertiary foramen, into the membranous part of the septum. Closure of the tertiary foramen is achieved by the end of the seventh week of development in the human heart, at around Carnegie stage 21, and by the end of E13.5 in the developing mouse (Figure 8a). It completes the process of ventricular septation. The septal attachments of the muscularised proximal cushions, by then, have been incorporated into the crest of the muscular ventricular septum. Subsequent to the completion of septation, it is no longer possible to recognise any anatomical boundary between the septal components, although the parts can be discerned on the basis of their second heart field lineage, with the parts derived from the cushions labelled by Isl1‐Cre (Figure 9).

### 3.5 Formation of the arterial valvar sinuses

**FIGURE 10:**
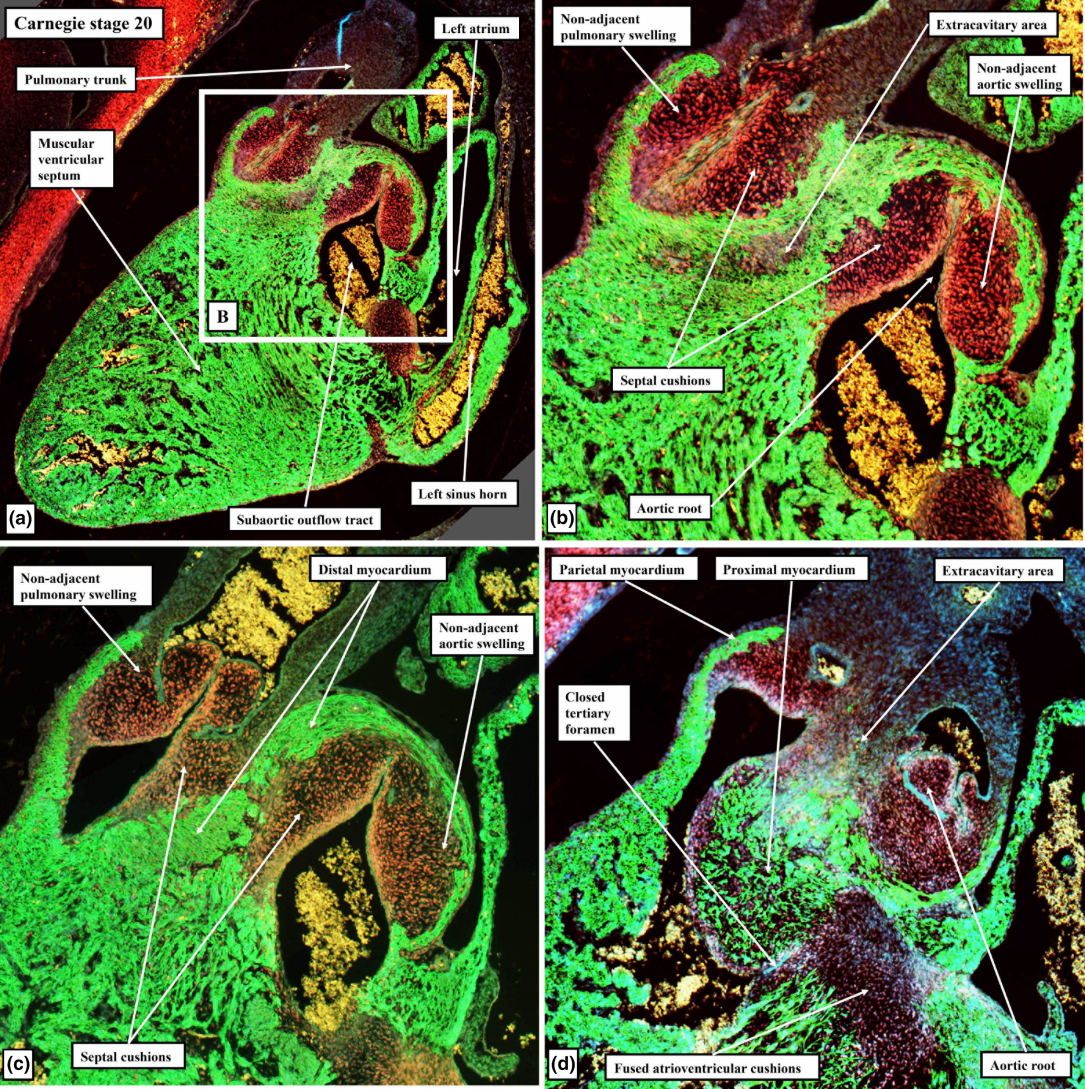
Serial sections from a CS20 human embryo cut in the sagittal plane, viewed from the left side, and immunostained to show the myocardium (cTnI, green) and the cushion tissue (Sox9; red). The area shown in the inset in panel (a) is enlarged to produce panel (b), with panels (c) and (d) then shown at comparable scale. The images show how the distal margins of the major cushions and intercalated valvar swelling are excavating to form the sinuses of the roots, with the major cushions now supported by the new myocardium formed by muscularisation of the distal cushions. Panel (d) shows how muscularisation of the proximal cushions has produced the infundibular sleeve, which itself has fused with the crest of the muscular ventricular septum to close the tertiary interventricular foramen. The extracavitary area between the roots can be seen in (b).

**FIGURE 11:**
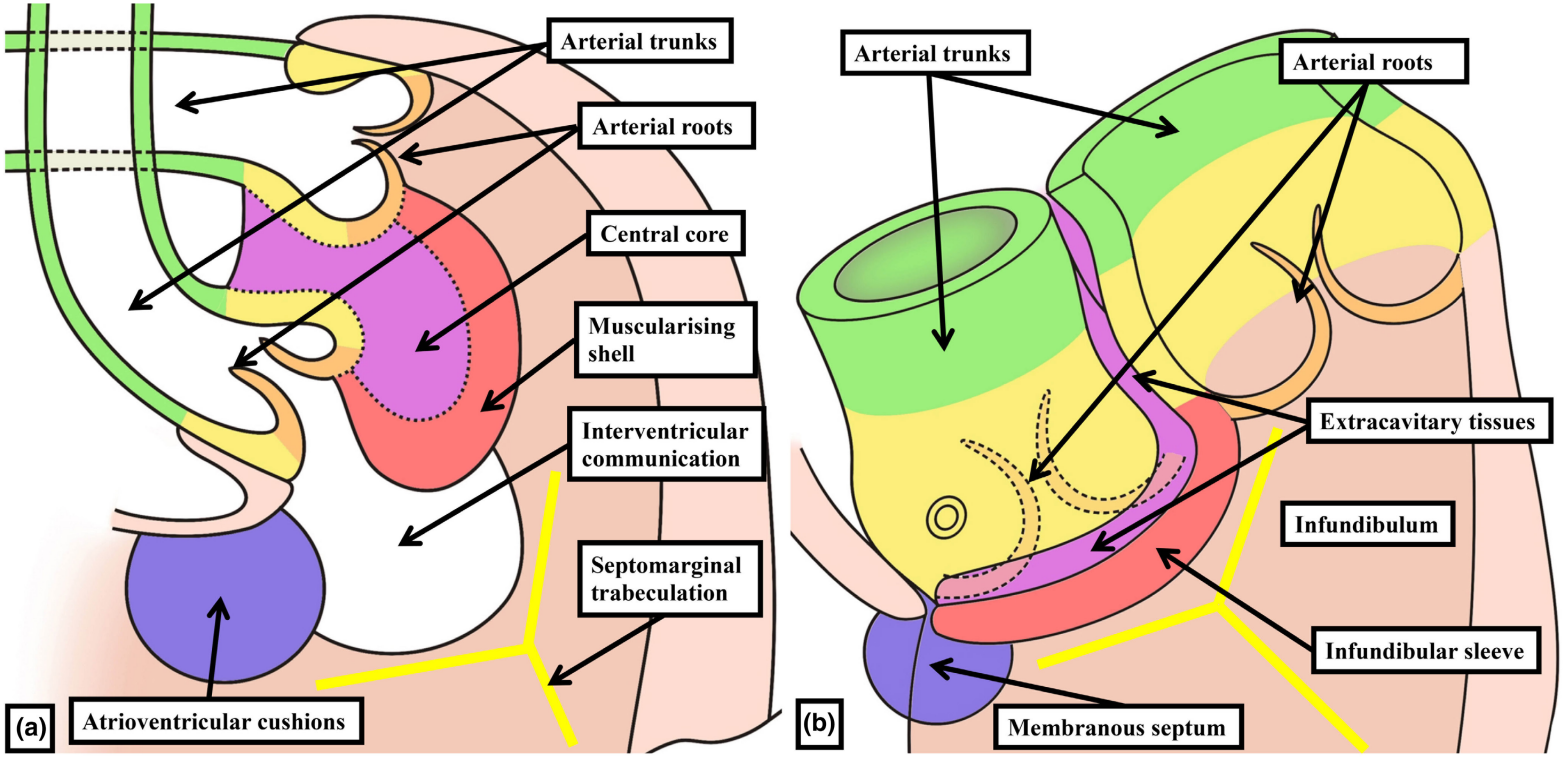
The drawings show the fate of the mesenchymal tissues that initially form the major cushions separating the middle and proximal parts of the outflow tract into the arterial roots and the ventricular outflow tracts. Panel (a) illustrates the muscularisation of the shell of the proximal cushion mass, with the central component failing to muscularise. As shown in panel (b), the core will be converted into an area of extracavitary tissue, comparable to the change shown in Figure 4 for the distal outflow tract. The newly formed extracavitary area becomes relatively reduced in its proportions compared to the extent of the infundibulum.

As development proceeds, further changes take place in the arrangement of the arterial roots. These changes involve the ongoing remodelling of the non‐myocardial tissues. In the stages described thus far, the entirety of the middle part of the outflow tract had been encased within a turret of myocardium. The integration of the non‐myocardial components into the middle part produces the walls of the sinuses of both arterial roots. As the sinus walls differentiate into smooth muscle cells and fibrous tissue (Richardson et al., 2018), they occupy areas created by excavation of the distal parietal margins of the major cushions and the intercalated valve swellings. The remodelling creates the pocket‐shaped cavities of the valvar sinuses. It is the excavated luminal components of the cushions and swellings that then form the semilunar leaflets of the developing valves (Figures 8b,c and 10). By these stages, the muscularised shell of the fused proximal cushions has been converted into the wall of the subpulmonary infundibulum. This now forms the outflow tract of the right ventricle (Figures 8b and 10). During these processes, the core of the fused cushion mass, which was initially substantial, has become markedly reduced in size. It remains as the extracavitary area of non‐myocardial tissue interposed between the wall of the subpulmonary infundibulum and the newly formed arterial walls of the sinuses of the aortic root (Figures 8b–d, 10 and 11). In the postnatal heart, most of the tissues are fibro‐adipose, but within the area it is also possible, in some hearts, to recognise the so‐called conus ligament, which is the remnant of the neural crest cells that formed the so‐called “whorl” (data not shown).

### 3.6 The relative movement of the distal myocardial border

**FIGURE 12:**
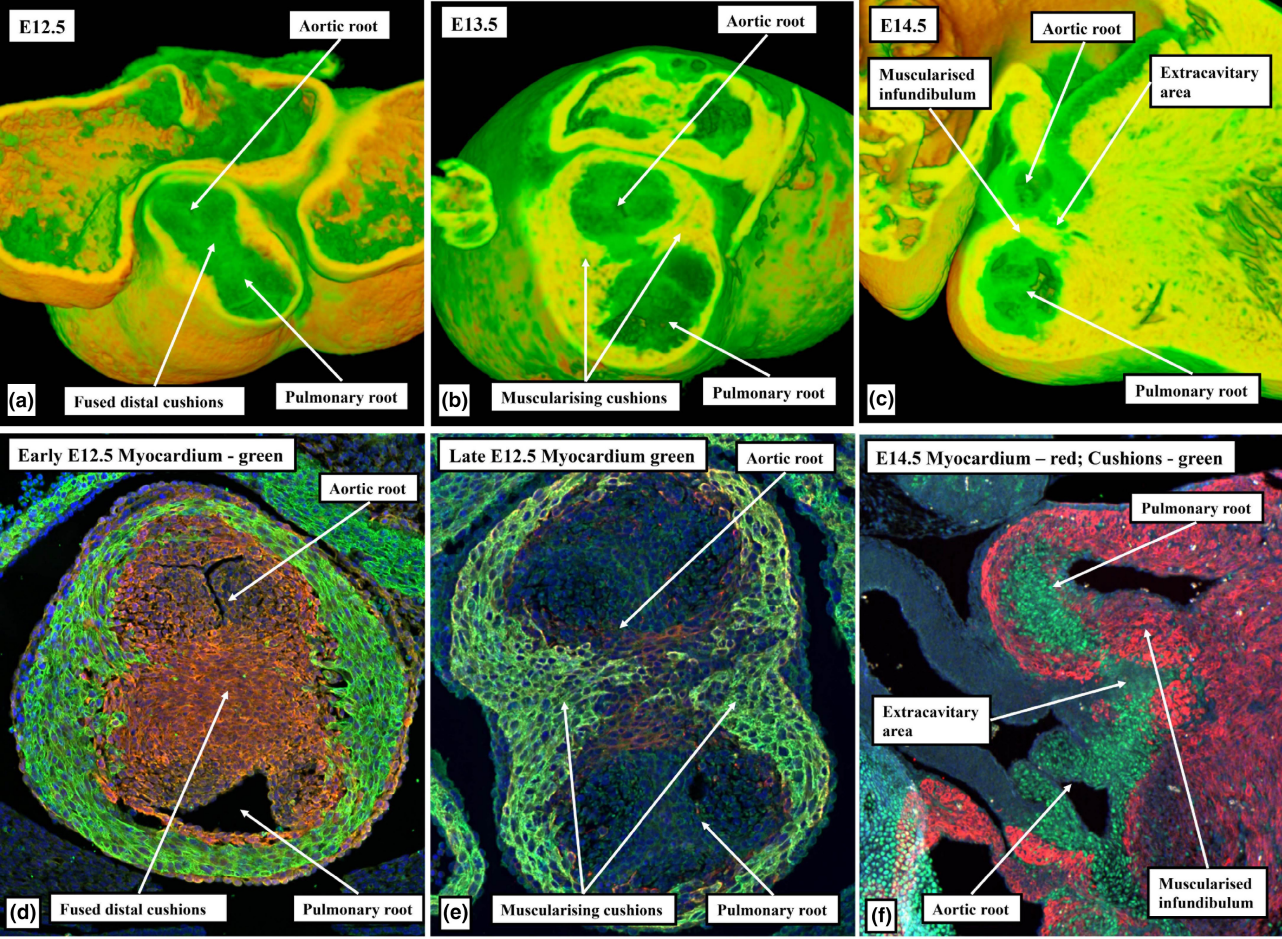
(a–c) Episcopic datasets are from XMLC2‐Cre mouse embryos (myocardium is yellow). (d–f) Immuno‐stained sections showing the myocardium (green in (d) and (e), red in (f)), cushion tissue (Sox9; red in (d), green in (f)) and smooth muscle cells (αSMA; red in (e)). Panels (a), (b), (d), and (e) are short axis sections through the middle of the middle part of the outflow tract. Panels (c) and (f) are oblique sections through the newly formed subpulmonary infundibulum. Panels (c) and (f) show its relationship to the aortic root, and the unstained extracavitary area between the infundibular sleeve and the aortic valvar sinuses, which are also non‐myocardial.

**FIGURE 13:**
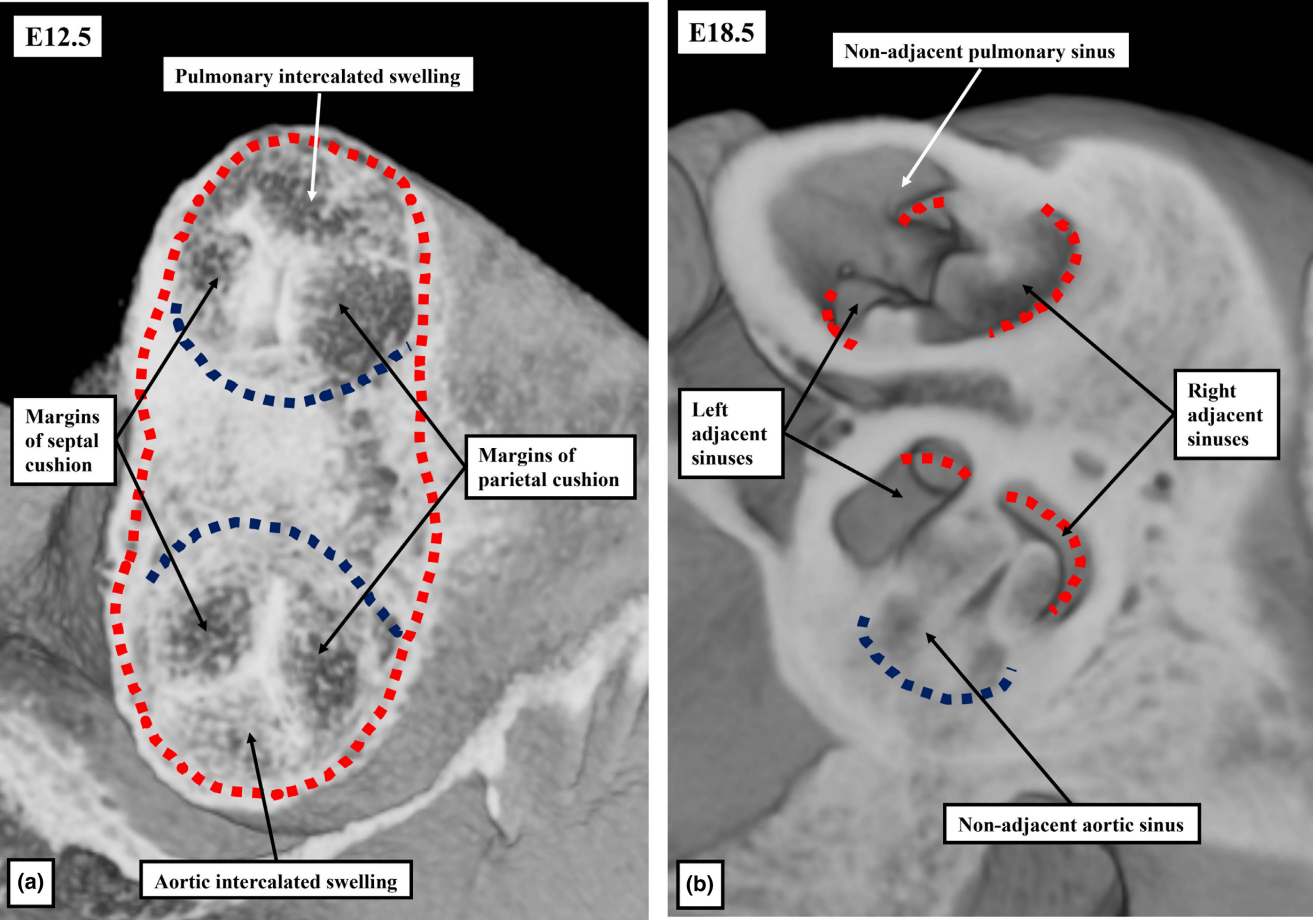
Optical sections from episcopic datasets from E12.5 and E18.5 mouse embryos. Both are across the short axis of the developing roots and are viewed from above. Panel (a) shows how the myocardium of the middle part of the outflow tract, shown by the red dashed line, and distinguished from the non‐myocardial tissues by its texture, surrounds both developing roots. The components destined to become the adjacent sinuses of the roots, however, shown by the black dashed lines, have no myocardial support at this stage. Panel (b) shows the arrangement just before birth, when there has been the formation of the valvar sinuses. Analysis of the histological sections reveals that areas of myocardium, with their sites shown by the red dashed lines, are now to be found at the bases of all the sinuses of the pulmonary root, but only the sinuses of the aortic root which give rise to the coronary arteries. The myocardium supporting the adjacent sinuses of both roots has been produced by muscularisation of the distal outflow cushions. The myocardium of the non‐adjacent aortic sinus has disappeared.

At the beginning of development, the distal myocardial border had been located at the margins of the pericardial cavity (Figure 1b). At the beginning of embryonic day 12.5 in the developing mouse, and at Carnegie stage 15 in the human, when the distal part of the outflow tract is now non‐myocardial, the distal myocardial border is located at the boundary between the distal and middle parts of the overall outflow tract (Figure 5a). At this stage, the persisting myocardial walls surround the developing arterial valves in the middle part of the outflow tract (Figure 12a). These myocardial walls, part of the original outflow tract, support both the parietal margins of the major cushions and the intercalated valve swellings, that then remodel to form the leaflets of the arterial valves. When initially fused to separate the developing arterial roots, the distal parts of the major cushions have no myocardial support, since the myocardial collar provides only circumferential support to the middle part of the outflow tract (Figure 13a). Individual support is required as the parts of the cushions that contribute to the aortic root are transferred to the left ventricle. It is provided by the muscularisation of the remaining parts of the distal components of the fused cushions that have not been remodelled to become the valvar leaflets (Figure 10a,b). As mentioned earlier, the muscularisation of the proximal parts of the fused cushions had produced the free‐standing subpulmonary infundibular sleeve. These changes can be recognised by CS23 in the human heart (Figure 9d), and by E14.5 in the developing mouse (Figure 10f). As the walls of the valvar sinuses are arterial, the distal myocardial boundary is now found more proximally, although the bases of the excavating cushions and swellings retain outer myocardial walls. The extent of the myocardium, however, varies within the different valvar sinuses. Thus, myocardium is found within the bases of the walls of all three sinuses of the pulmonary root, but only within the walls of the sinuses of the aortic root which give rise to the coronary arteries (Figure 13b).

### 3.7 Formation of the walls of the valvar sinuses

**FIGURE 14:**
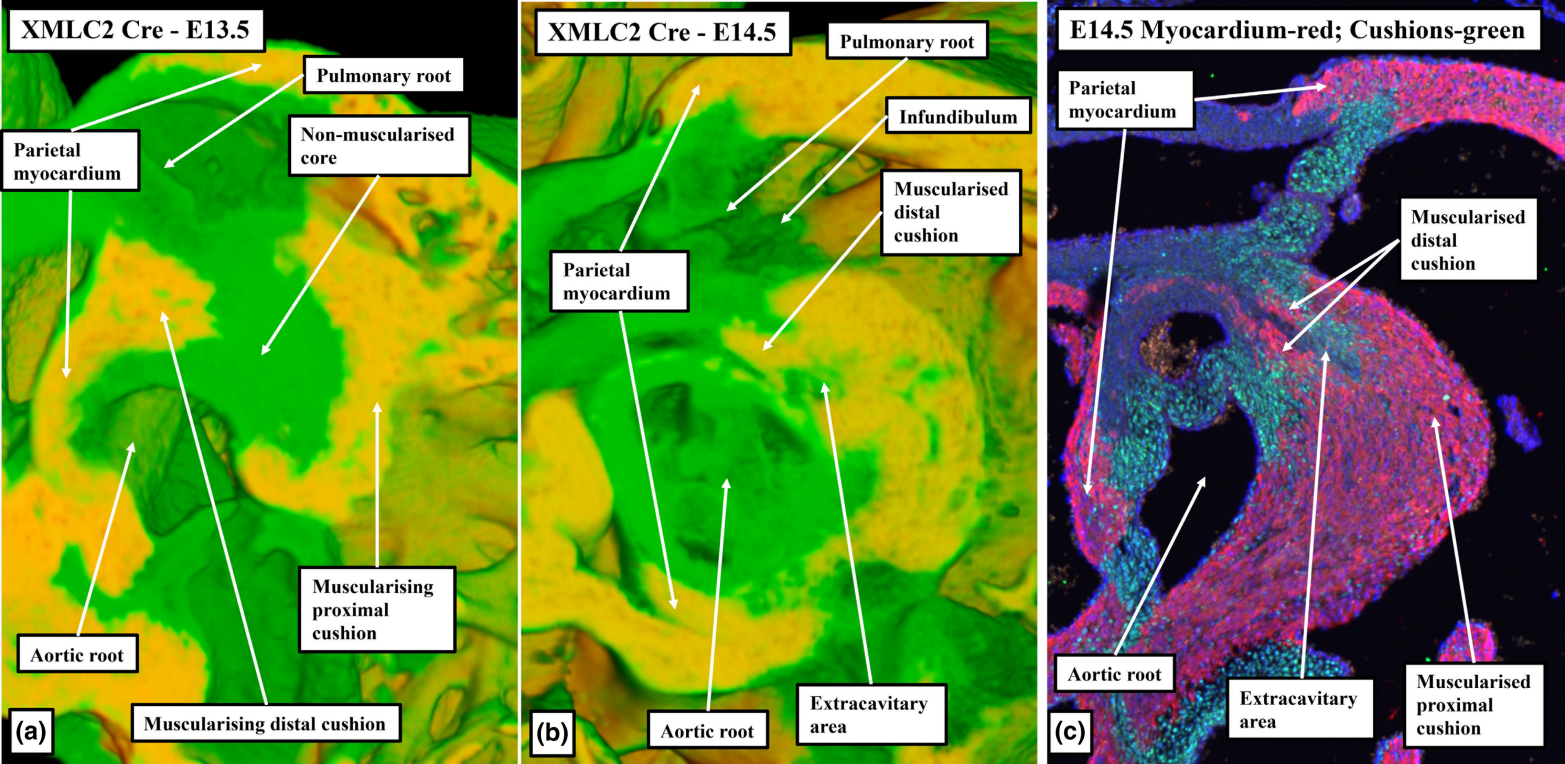
Panels (a) and (b) are from episcopic datasets from XMLC2‐Cre embryos at embryonic days 13.5 (panel a) and 14.5 (panel b), with the myocardium shown in yellow. The sections are taken in the sagittal plane through the fused cushion mass, showing the formation of the right ventricular infundibulum, and its separation by the core of the tissue mass from the newly formed sinuses of the aortic root. Panel (c) is a similar section immunostained with myocardium coloured red and cushion tissue green.

**FIGURE 15:**
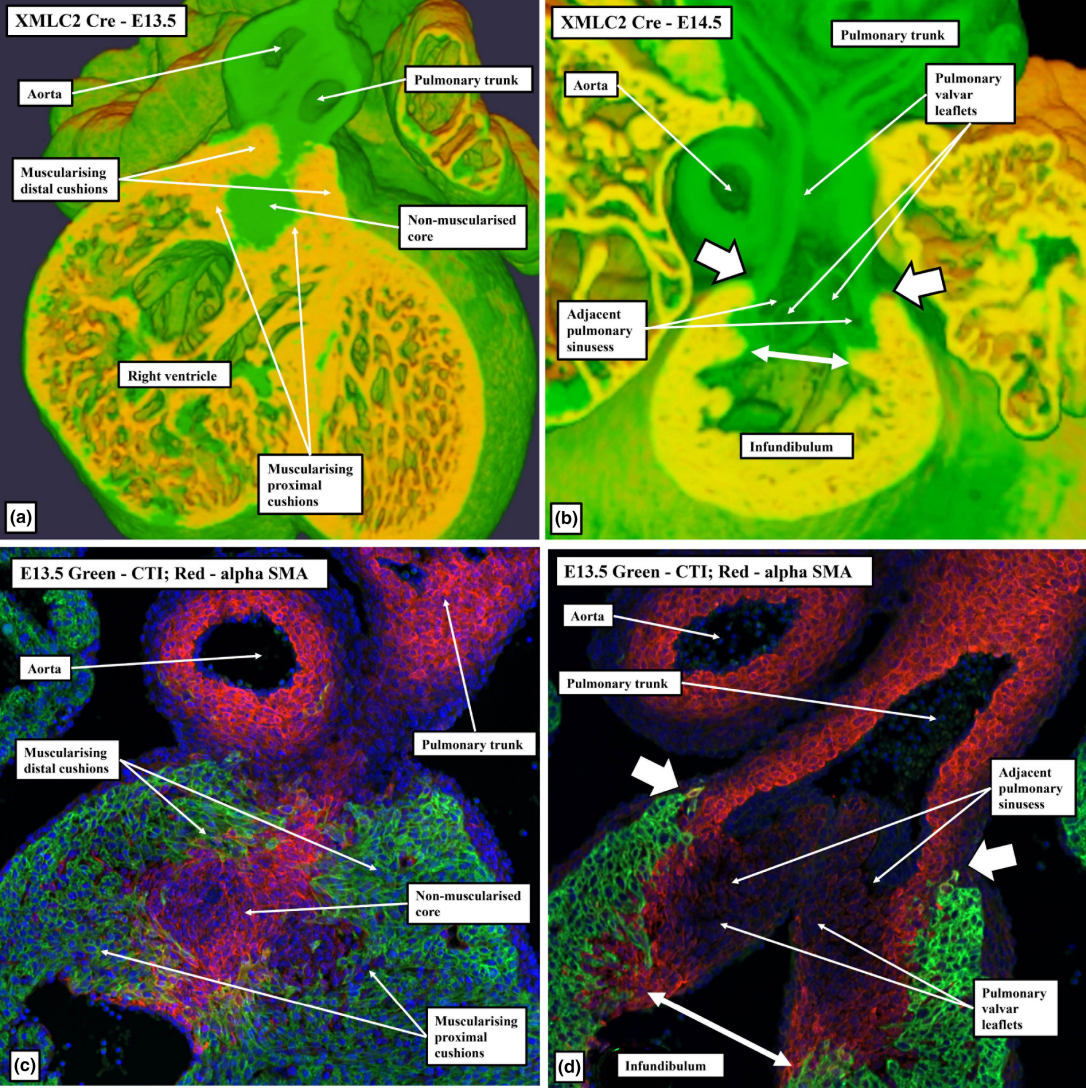
(a, b) Images from E13.5 and E14.5 episcopic datasets prepared from XMLC2‐Cre+ mouse embryos, with myocardium shown yellow. (c, d) Comparable E13.5 mouse sections immunostained to show the myocardium (cTnI; green) and smooth muscle cells (αSMA; red). Panels (a) and (c) are in the frontal plane through the middle of the fused cushions and show muscularisation of both the distal and proximal parts of the cushions, with the core remaining non‐myocardial. Panels (b) and (d) are frontal sections through the pulmonary root showing the myocardium supporting the bases of the two adjacent sinuses of the pulmonary root. Note that the distal extent of the myocardial border (white arrows with black borders) is well distal to the proximal extent of the semilunar valvar leaflets (white double‐headed arrows). It is the muscularised distal cushions that continue to support the remodelling leaflet.

**FIGURE 16:**
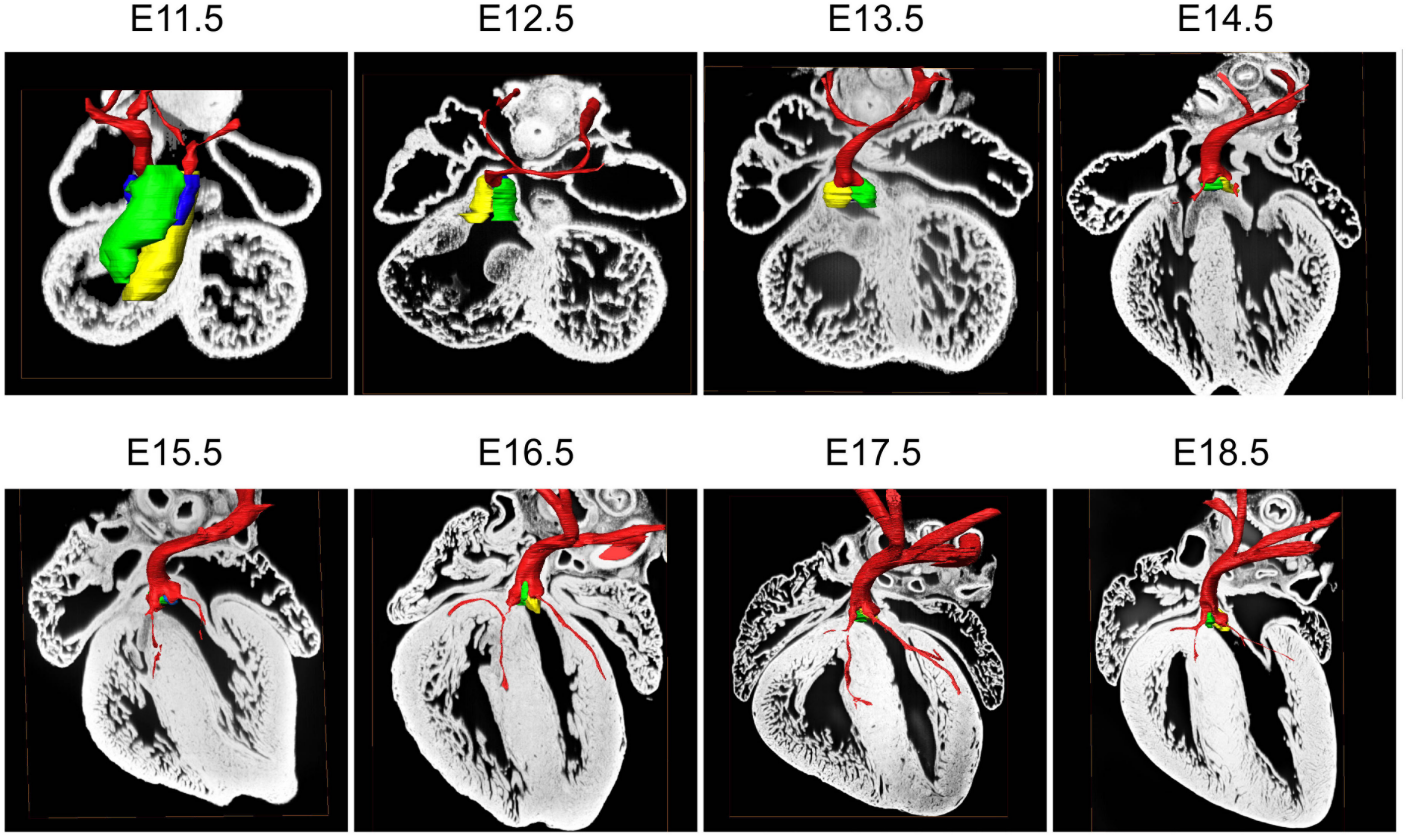
Reconstructions from episcopic datasets from a series of mouse embryos from E11.5 to E18.5, showing the formation of the aortic root from the distal parts of the outflow cushions, and as shown at higher magnification, its eventual incorporation from E15.5 into the infero‐septal recess of the left ventricle. The colours are as shown in Figures 2 and 4, with red showing the newly formed sinuses of the aortic root and the coronary arteries.

**FIGURE 17:**
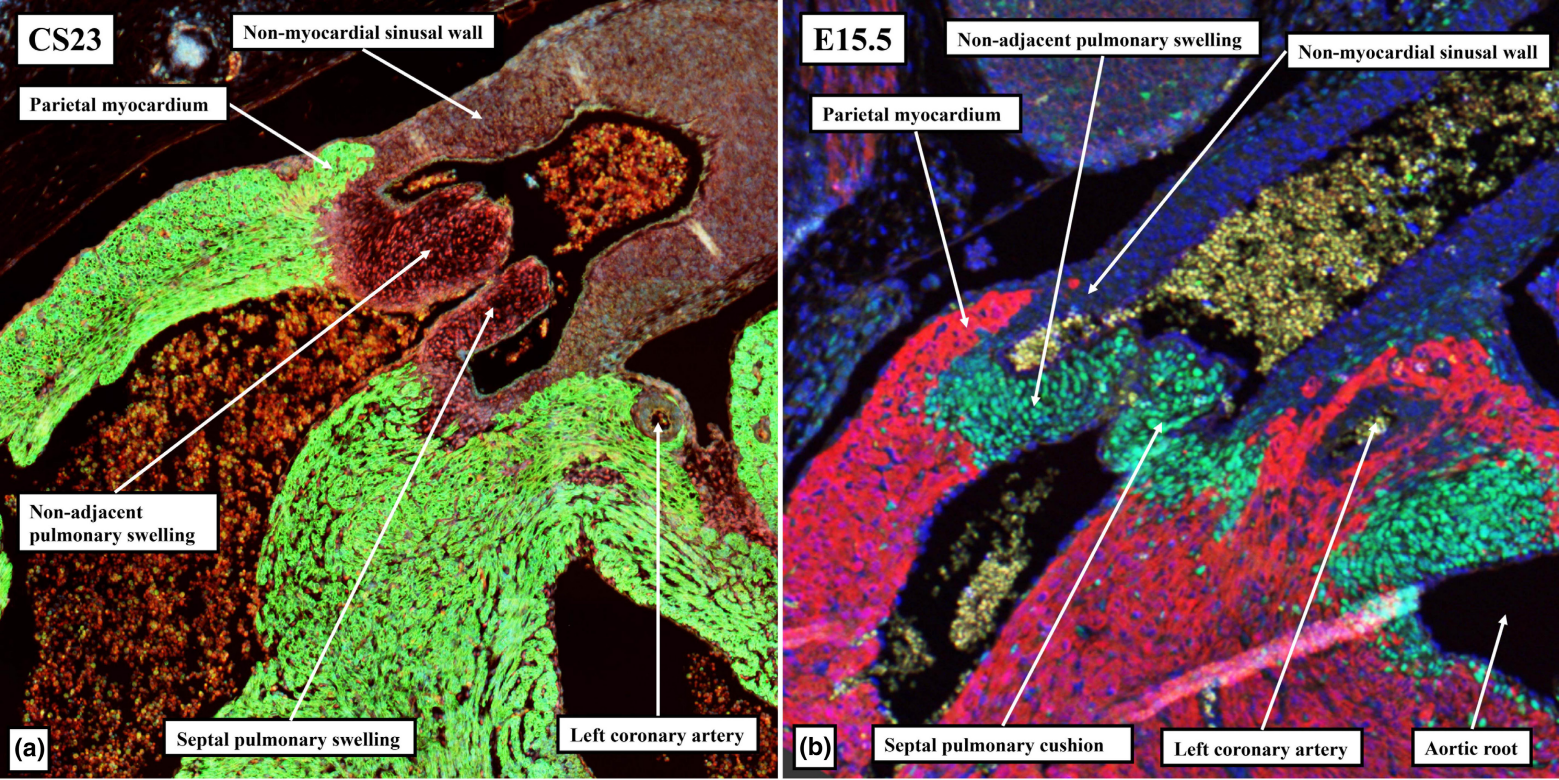
CS23 human embryo (a) and E15.5 mouse embryo (b) immunostained to label the myocardium (cTnI; green in (a), red in (b)) and cushion tissue (Sox9; red in (a), green in (b)). Excavation of the distal margins of the intercalated pulmonary valvar swelling and the septal pulmonary major cushion has produced the leaflets of the pulmonary root, with the excavating leaflets supported by the outflow myocardium. The leaflet excavated from the intercalated valve swelling is supported by the initial parietal myocardium of the middle part of the outflow tract, whereas the opposing leaflet is supported by the myocardium formed by muscularision of the distal septal pulmonary cushion. The newly formed myocardium is fully incorporated into the crest of the muscular septum.

When it first became possible to recognise the valvar primordiums, during E12.5 in the mouse, and at CS17 in the human, there were no valvar sinuses (Figure 11a,b). At these initial stages, the distal surfaces of the cushions and intercalated valve swellings were confluent with the distal myocardial border (Figures 5e and 6d). Eventually, with the exception of the non‐coronary sinus of the aortic root, myocardium will still be found surrounding the bases of the walls of all the remaining sinuses; albeit with the myocardial wall lined by a luminal fibrous layer derived from the cushions and the swellings (Richardson et al., 2018). Myocardium, however, also initially supported the wall of the developing non‐coronary aortic sinus (Figures 9 and 10). This myocardium was part of the initial myocardial wall of the middle part of the outflow tract itself. The myocardium that supports the developing leaflets of the adjacent left and right sinuses of both arterial roots, in contrast, had been formed by the muscularisation of parts of the distal major outflow cushions (Figures 14 and 15). The myocardium supporting the wall of the non‐coronary sinus of the aortic root in both the human and mouse hearts eventually disappears in its entirety (data not shown). It is the disappearance of this myocardium that produces the fibrous continuity between the leaflets of the mitral and aortic valves found at the base of the left ventricle subsequent to birth. During the overall process of remodelling, the aortic root itself becomes “wedged” into the infero‐septal recess of the left ventricle between the aortic, or anterior, leaflet of the mitral valve and the inferior part of the ventricular septum (Figure 16). With the exclusion of the non‐coronary sinus, the distal myocardial border continues to lie distal to the level of the basal attachment of the semilunar valvar leaflets. The temporal change in the location of the boundary is best assessed in the non‐adjacent sinus of the pulmonary root (compare Figures 15b,d and 17). The luminal components of the cushions and swellings have then completed their own remodelling to form the valvar leaflets, also producing the fibrous endocardial layer that lines the myocardial components of the walls of the sinuses.

## 4 SUMMARY AND CONCLUSIONS

The separation, during development, of the outflow tracts from a tube with exclusively myocardial walls to the arrangement of systemic and pulmonary channels, each with its own discrete walls, involves the appearance of the intrapericardial arterial trunks and the formation of the arterial roots. This then leaves the proximal parts of the embryonic outflow tract to be incorporated as the definitive ventricular outflow tracts. During these periods, the relative lengths of the outflow tracts with myocardial walls have decreased significantly. At the same time, new myocardium has been formed by muscularisation of parts of the cushions that initially fused to separate the common lumen into the pulmonary and aortic channels (van den Hoff et al., 1999; van den Hoff & Wessels, 2020). This new myocardium, proximally, will produce part of the free‐standing pulmonary infundibular sleeve. Distally, it persists as the myocardial crescents found within the bases of the walls of the adjacent sinuses of both the aortic and pulmonary roots (Mori et al., 2019; Toh et al., 2020). The major outflow cushions, along with the protrusion from the dorsal wall of the aortic sac, initially created a septum within the common outflow channel (Anderson et al., 2012). As each of the outflow tracts becomes an individual entity, however, it is no longer possible to find any septal structures between them (Anderson, Tretter, et al., 2019). This loss of septal identity occurs concomitant with the remodelling of the arterial walls and with the development of the free‐standing subpulmonary infundibular sleeve. With these changes, the central cores of the initial mesenchymal septal tissues become converted into the tissues that now interpose as extracavitary areas between the separate walled channels.

During these processes, the attachment of the muscularised proximal septal cushion has been incorporated within the crest of the muscular ventricular septum. The assimilation is sufficiently complete that it becomes impossible, using anatomical dissection, to distinguish between the outflow myocardium and the remainder of the muscular ventricular septum. The components can be recognised, nonetheless, on the basis of their lineages, at least in the mouse. This incorporation into the aortic root and into the crest of the ventricular septum, of tissues derived from the second heart field has important clinical significance. The myocardial crescents in both arterial roots are the substrates for outflow tract arrhythmias (Anderson, Mohun, et al., 2019). The extent of outflow myocardium incorporated into the septal crest also determines the adjacency of the superior fascicle of the left bundle branch to the nadir of the right coronary aortic valvar leaflet. Recognition of this feature can help those performing transcatheter replacement of the aortic valve avoid damage to the left bundle branch (Macías et al., 2022).

The overall changes seen during development are also pertinent to descriptions of the outflow tract in the setting of congenital cardiac malformations. At present, it is common for paediatric cardiologists to describe malformations involving the outflow tracts as being “conotruncal.” As we emphasised in our introduction, the bicuspid aortic valve, the commonest congenital cardiac lesion (Roberts, 1984), which is formed between the distal and proximal parts of the outflow tract, is not conventionally considered a “conotruncal” malformation (Van Praagh, 2016). Common arterial trunk, furthermore, is still frequently described as a “persistent truncus.” In this regard, it was the distal part of the outflow tract that Kramer nominated as the truncus (Kramer, 1942). Yet it is the commonality of the ventriculo‐arterial junction, located between the middle and proximal parts of the outflow tract, which is the phenotypic feature of the lesion (Spicer & Steffensen, 2020). All these aspects point to the superiority of accounting for the outflow tract in terms of three, rather than two, components. These are the intrapericardial arterial trunks, the arterial roots, and the subvalvar ventricular outflow tracts. The changes we have now described for the distal, middle and proximal parts of the developing outflow tract can be correlated accurately with the appearance of these three definitive components. When first formed, however, the outflow tract has only proximal and distal parts, each having myocardial walls. It is the appearance of the non‐myocardial arterial trunks that heralds the change from the bipartite to the tripartite configuration. The myocardial component initially presents distally and then supports and surrounds the middle part of the definitive outflow tract.

Our current account seeks to explain only anatomical changes, although these are supported by the findings showing the specific makeup of the different tissues (Eley et al., 2018; Henderson et al., 2020; Richardson et al., 2018). The underlying three‐dimensional anatomical changes have also received attention (Mohun & Anderson, 2020). More now needs to be done to determine the precise mechanics of the changes that, anatomically, underscore the proximal change in the position of the distal myocardial border when assessed relative to the boundaries of the pericardial cavity. It may be that the cardiomyocytes are simply translocated closer to the ventricular base by the differential growth of the myocardial and non‐myocardial tissues. These mechanisms, nonetheless, require further investigation, as does the mechanism of removal of the myocardium found supporting the non‐adjacent sinus of the aortic valve. Additional work is also required to explain the precise mechanisms underscoring the changes observed in the cushions and swellings as they remodel to form, in part, the arterial valvar leaflets, but also the fibrous tissues that line the myocardial bases of the walls of the valvar sinuses. These mechanistic changes will only properly be appreciated when account is taken of the multiple anatomical changes currently described. In many ways, our current investigation serves to emphasise the validity of the statement made over 80 years ago by Kramer (1942). Many of the problems that have remained subsequent to his study have, indeed, related directly to the rapidly occurring shapes and locations observed during the development of the outflow tract. We hope our review has clarified the significance of these changes.

## Data Availability

Not applicable.
